# Could lengthening minocycline therapy better treat early syphilis?

**DOI:** 10.1097/MD.0000000000005773

**Published:** 2016-12-30

**Authors:** Li-Li Shao, Rui Guo, Wei-Jie Shi, Yuan-Jun Liu, Bin Feng, Long Han, Quan-Zhong Liu

**Affiliations:** Department of Dermatovenereology, Tianjin Medical University General Hospital, Tianjin, China.

**Keywords:** China, minocycline, penicillin, serological cure rate, syphilis

## Abstract

Syphilis is a sexually transmitted disease caused by *Treponema pallidum*. Minocycline, a representative tetracycline derivative, has the greatest antimicrobial activity among all tetracyclines. There are few reports about treating syphilis with minocycline because there is a lack of efficacy data from controlled trials. We compared the rates of serological cure in patients with early syphilis who were treated with minocycline or benzathine penicillin G (BPG).

During the study period, a total of 40 syphilis patients received the BPG treatment, which was a single intramuscular dose of 2.4 million units of BPG, and 156 patients were treated with minocycline; 77 patients were placed in the 2-week, standard minocycline therapy group and received 100 mg of minocycline orally, twice daily for 14 days, and 79 patients were placed in the 4-week, lengthened minocycline therapy group and received 100 mg of minocycline orally, twice daily for 28 days. The outcome of interest was the rate of serological cure in these patients.

At the end of the 2-year follow-up, the serological cure rate of the 4-week, lengthened minocycline therapy group (87.34%) was higher than that of both the 2-week, standard minocycline therapy group (72.73%) and the BPG treatment group (77.50%). In addition, the curative effect of the 4-week, lengthened minocycline therapy was significantly greater than that of the 2-week, standard minocycline therapy in patients who were aged >40 years; exhibited an initial rapid plasma reagin titer ≥1: 32; or exhibited secondary syphilis (*P* = 0.000, 0.008, 0.000; <0.05).

Minocycline appears to be an effective agent for treating early syphilis, especially when applied as a 4-week, lengthened therapy.

## 1 Introduction

Syphilis is a relatively common, systemic disease caused by *Treponema pallidum* (*Tp*). The World Health Organization (WHO) estimated that approximately 10.6 million incident cases of syphilis occur worldwide each year.^[1]^ In recent decades, syphilis has made a dramatic resurgence in China.^[2,3]^ In 1999, the reported incidence rate of infectious syphilis in China was 6.5 per 100,000, but by 2009, this rate increased by approximately 4-fold to 23.1 per 100,000.^[4]^ Currently, syphilis is among the top 3 reported communicable diseases in China.^[2]^ Because syphilis patients are the only sources of syphilis transmission, the early diagnosis and treatment of syphilis not only can effectively control the spread of syphilis but also are of great significance for health planning and management.^[3,5]^

The cure rate of syphilis has reportedly ranged from 78.5% to 95% in recent years.^[6,7]^ However, after undergoing the recommended treatment regimen, many syphilis patients still exhibit reactive serum rapid plasma reagin (RPR) test results or clinical symptoms. Longer therapeutic courses or higher drug dosages may be feasible approaches toward improving the treatment efficacy and cure rate of syphilis. Unfortunately, there have been few reports about this type of research until now. In the “Sexually Transmitted Diseases Treatment Guidelines (2015 Edition)” of the US Centers for Disease Control and Prevention (CDC) and the 2014 European guidelines on the management of syphilis,^[8,9]^ long-acting penicillin G (ie, benzathine penicillin G [BPG]) administered parenterally is the only first-line regimen recommended for early syphilis. In some cases, patients cannot tolerate this therapy because they exhibit positive skin test results to penicillin G, indicating an allergic reaction. Thus, physicians often choose alternative clinical regimens for these patients. Although there are limited data supporting the use of alternatives to penicillin for treating primary and secondary syphilis, some studies have reported that doxycycline and tetracycline may be reasonable substitutes.^[10]^ Minocycline is a representative tetracycline derivative that has the greatest antimicrobial activity among all tetracyclines, and also better oral bioavailability, fewer gastrointestinal side effects, and lower clinical resistance. Minocycline has a long half-life and can achieve sustainable and stable plasma concentrations in vivo. In China, minocycline and BPG treatments for early syphilis are reportedly not significantly different in terms of sero-resistance and sero-relapse.^[11]^ Thus, minocycline may be a preferable alternative for penicillin-allergic patients or individuals who are unwilling to accept injection therapy.

This study retrospectively analyzed syphilis patients in the Tianjin Medical University General Hospital sexually transmitted disease (STD) outpatient clinic who were treated with minocycline or BPG. According to the different therapeutic durations, patients who were treated with minocycline treatment were divided into 2 groups: the 2-week, standard therapy group and the 4-week, lengthened therapy group. The follow-up period lasted for more than 2 years, and the outcome of interest was the serological cure rate after these patients were completely clinically cured.

## 2 Methods

### 2.1 Study participants

The participants were syphilis patients who visited the STD clinic of the Tianjin Medical University General Hospital, China, between January 2011 and December 2013. This study was approved by the Ethics Committee of the Tianjin Medical University General Hospital. The need for informed consent was waived by the Ethics Committee because the study was an observational, retrospective study using a database from which the patients’ identifying information had been removed.

### 2.2 Data collection

Among these 875 cases, 137 were primary syphilis cases, 193 were secondary syphilis cases, 4 were late syphilis cases, 3 were congenital syphilis cases, and 538 were latent syphilis cases. All patients were diagnosed with different stages of syphilis according to the national Centers for Disease Control diagnostic standards.

The inclusion criteria were as follows:

(1) The patients must have had a first-time diagnosis of early syphilis (in the primary, secondary, or early latent stages).
(2) The patients must have had at least 2 serological titers within 24 months, with 1 titer at or around the date of treatment, that is, baseline titer.
(3) The patients must have had regular follow-ups at 3, 6, 9, 12, 18, and 24 months posttreatment to review their clinical symptoms and serum RPR titers.
(4) The patients must have received a recommended regimen based on the national Sexually Transmitted Infections Guidelines even if the syphilis patients were coinfected with other STDs, including *Ureaplasma urealyticum*, *Mycoplasma hominis*, *Chlamydia trachomatis*, or *Neisseria gonorrhoeae*.

Patients were excluded as per the following criteria:

(1) The patients were diagnosed to be infected with human immunodeficiency virus (HIV) or pregnant.
(2) The patients did not have follow-up data or had a total follow-up period <2 years.
(3) The patients did not receive a recommended regimen based on the national Sexually Transmitted Infections Guidelines.

The recommended regimens consisted of the following:

(1) Two-week, standard minocycline therapy: 100 mg of minocycline orally, twice daily, for 14 days (ie, the MINO-2w group).
(2) Four-week, lengthened minocycline therapy: 100 mg of minocycline orally, twice daily, for 28 days (ie, the MINO-4w group).
(3) BPG treatment: a single intramuscular dose of 2.4 million units of BPG (ie, the BPG group).

**Figure 1:**
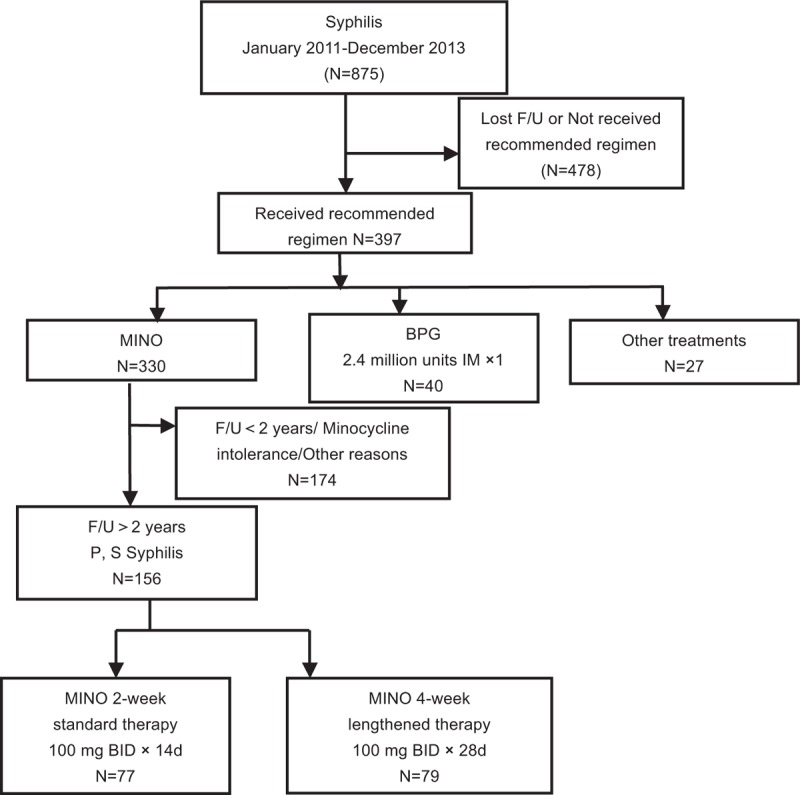
Case selection of benzathine penicillin G (BPG)-treated patients and minocycline (MINO)-treated patients reported in the Tianjin Medical University General Hospital, China, 2011–2013.

One hundred and ninety-six cases of the 875 cases were included in the final study sample. A total of 77, 79, and 40 patients were included in the MINO-2w group, the MINO-4w group, and the BPG group, respectively (Fig. 1).

### 2.3 Variables of interest and definitions

As a routine in our STD clinic, all the patients were asked to review their clinical symptoms and serum RPR titers every 3 months for 2 years after treatment. Patients who had persistent or recurring signs or symptoms, and patients with at least a 4-fold increase in their nontreponemal test titer persisting for >2 weeks likely experienced treatment failure or were re-infected. The syphilis patients were considered to be clinically cured if they exhibited no clinical manifestations of the disease. In addition, syphilis patients whose nontreponemal (ie, Venereal Disease Research Laboratory [VDRL] or RPR) test results reverted from reactive to nonreactive within 2 years after treatment were defined as being serologically cured. The US CDC states that the VDRL and RPR are equally valid assays, but the quantitative results from the 2 tests cannot be compared directly because RPR titers frequently are slightly higher than VDRL titers.

In this study, the outcome of interest was the rate of serological cure after these patients were completely clinically cured, that is, the number of patients whose RPR titers became nonreactive after the disappearance of clinical manifestations of syphilis. The clinical therapeutic effect depended on the rate of serological cure.

### 2.4 Statistical analysis

Data were analyzed using SPSS for Windows, version 19.0. Pearson chi-square test was used to compare differences in categorical variables; *P* values <0.05 were considered to represent statistical significance.

## 3 Results

A total of 156 syphilis patients received a minocycline treatment, and they ranged in age from 16 to 79 years. Among these 156 cases, 71 (45.51%) patients were male and 85 (54.49%) patients were female. This study included comparative analyses among sex, age, number of sex partners, initial RPR titers, disease stage, and the presence of coinfection with other STDs (Table 1).

**Table 1:**
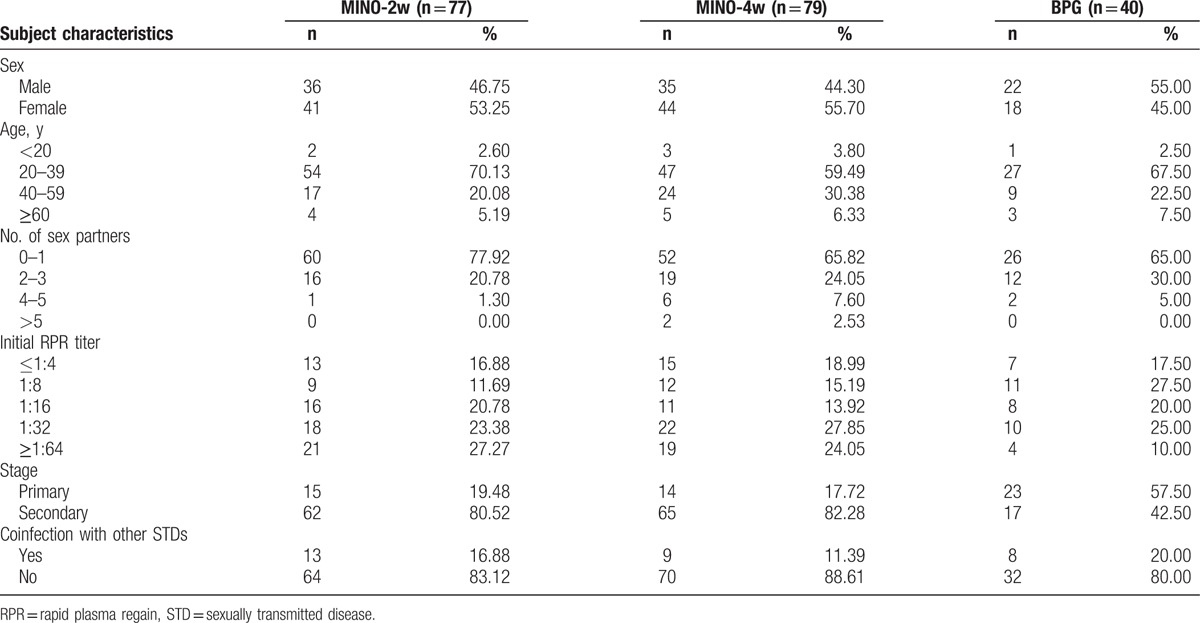
Baseline characteristics of early syphilis patients by treatment group.

**Figure 2:**
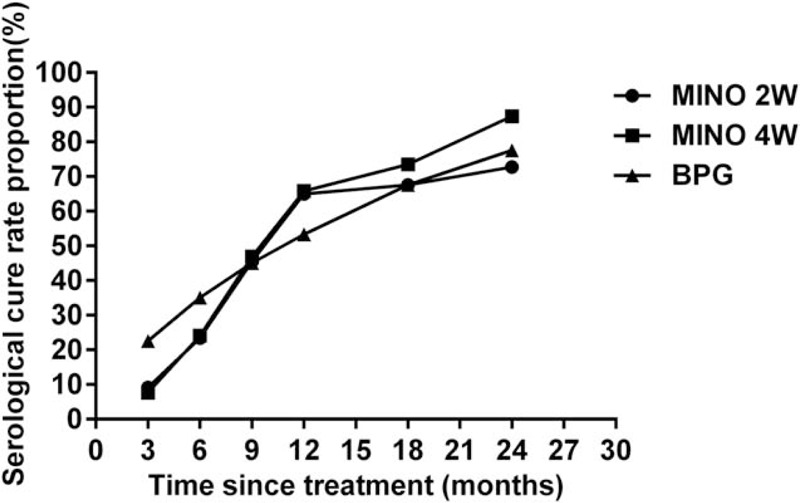
Serological cure rate proportions in the MINO-2w, MINO-4w, and BPG groups over time from 3 to 24 months. Over the 1-year follow-up, the serological cure rate in the MINO-2w group was statistically similar to that in the MINO-4w group (*P* = 0.907). After the 2-year follow-up, the serological cure rate in the MINO-4w group was significantly higher than that in the MINO-2w group (*P* = 0.022). In addition, the serological cure rate in the BPG treatment group was statistically similar to the rates observed in the MINO-2w and MINO-4w groups (*p* = 0.575 and 0.166, respectively).

In this study, after 1 year, the serological cure rate in the MINO-2w group was 64.93%, and that in the MINO-4w group was 65.82%; these rates were statistically similar. After 2 years, a serological cure rate of 87.34% (69/79) was observed in the MINO-4w group compared with 72.73% (56/77) in the MINO-2w group; these rates were significantly different (*P* = 0.022). The serological cure rate in the BPG treatment group was 77.50% (31/40), which was statistically similar to the rates observed in the MINO-2w and MINO-4w groups (*P* = 0.575 and 0.166, respectively) (Fig. 2).

Significance differences in serological cure rate were observed between the MINO-4w and MINO-2w groups in patients who were aged >40 years; exhibited an initial RPR titer ≥1:32; or exhibited secondary syphilis (*P* = 0.000, 0.008, and 0.000; <0.05). In these circumstances, the curative effect of the 4-week, lengthened minocycline therapy was obviously greater than that of the 2-week, standard minocycline therapy (Table 2).

**Table 2:**
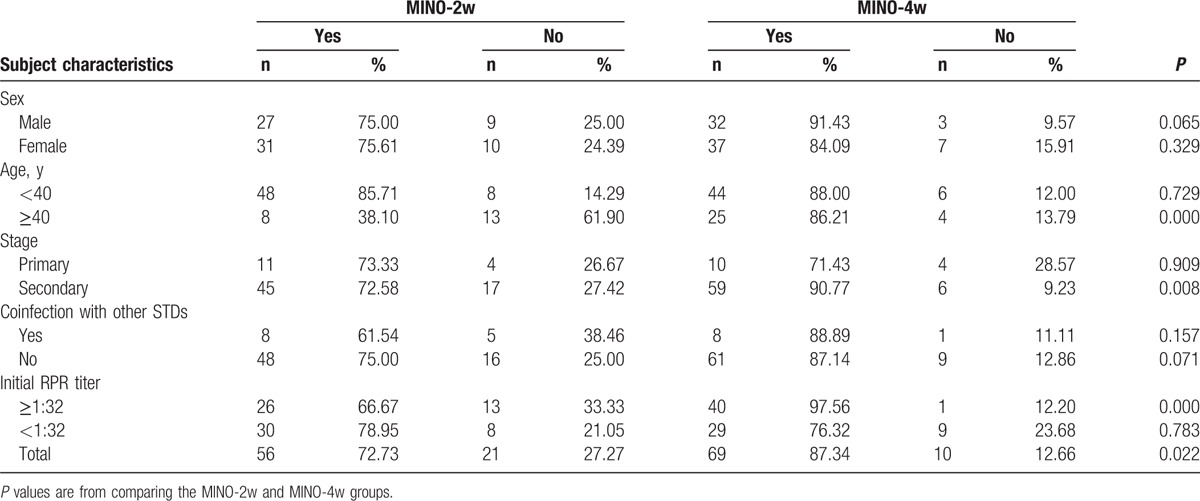
Number of patients with different characteristics who were or were not serologically cured by the 2-week, standard minocycline therapy (MINO-2w) and the 4-week, lengthened minocycline therapy (MINO-4w).

## 4 Discussion

### 4.1 Factors associated with serological cure

Although previous studies have investigated the relationships of patient characteristics, such as sex, age, disease stage, baseline RPR titer, and HIV coinfection status, with the serological cure rate of syphilis, no agreements have been reached.^[12,13]^ Tong et al^[14]^ found that patients who were >40 years old had a relatively lower probability of achieving a serological cure than did patients who were <23 years old. A study by Seña et al^[12]^ also concluded that an age of <30 years was associated with an increased likelihood of serological cure. Older individuals may have insufficient serological responses to syphilis therapy because of immune system senescence or immunosuppression.^[8,15,16]^ In contrast, Romanowski et al^[13]^ reported results that did not support a relevant relationship between age and serological cure status. Some studies have indicated that earlier diagnoses and treatments, and higher baseline RPR titers would contribute to higher serological cure rates of syphilis.^[12,13,17,18]^ Rolfs et al^[19]^ and Ghanem et al^[20]^ found that HIV-coinfected patients had a higher serological failure rate and required more time to achieve a serological response. By analyzing the common characteristics in patients who were not cured, our study revealed that in older patients, longer or later treatments of this disease may have lower serological cure rates. Meanwhile, sex or coinfection with other STDs seemed to have no influences on the cure rate. We speculate that these results may be related to lower immunity in the older patients and higher levels of *Tp* in longer courses or later treatments of syphilis. All these factors may render the elimination of *Tp* difficult. Regrettably, due to the limited amount of related research and the small sample size, such hypotheses remain to be confirmed.

A previous study by Chen^[21]^ found that the drug type, dosage, and route of administration also affected the serological cure rate. It is particularly important to select the treatment dosage and duration according to the stage and clinical manifestations of the disease. In the past, some specialists recommended that longer courses or larger dosages of syphilis treatments may be helpful for increasing the serological cure rate.^[22,23]^ Tittes et al^[24]^ found that the serological cure rate in patients treated with a single dose of BPG was significantly better than that in patients who received an enhanced therapy of 3 doses for primary and secondary syphilis. Penicillin G has been the first-line treatment for syphilis for a long time in all global guidelines; however, we cannot ignore that the prevalence of penicillin allergy reported in the United States is approximately 8% to 10%, and might be even higher in developing countries.^[25,26]^ In this situation, US doctors recommend desensitization in consultation with a specialist, whereas Chinese doctors choose alternative, inexpensive drugs that may easily be accepted by patients to avoid the application of penicillin. However, the intense controversy regarding alternatives to penicillin G continues because of a lack of data and research.

Doxycycline (100 mg orally, twice daily for 14 days) and tetracycline (500 mg, 4 times daily for 14 days) have long been used as the recommended second-line treatments for penicillin-allergic patients in many countries. Furthermore, compliance with the doxycycline treatment seemed to be better than that with tetracycline because of the fewer gastrointestinal side effects and lower dosage. Wong et al^[27]^ compared the serological treatment success rate of patients with primary syphilis treated with doxycycline/tetracycline with that of patients treated with a single dose of BPG; the results showed that doxycycline/tetracycline had a high serological treatment success rate similar to that of penicillin. Li and Zheng^[10]^ also reported results demonstrating that doxycycline/tetracycline and BPG had similar serological treatment success rates when used to treat early syphilis. Similar results from comparing doxycycline with BPG for the treatment of early syphilis have been reported by Ghanem et al.^[20]^ In addition, some cases have indicated that the serological response rates to doxycycline and BPG therapy were similar in HIV-infected patients with early syphilis after 6 and 12 months.

### 4.2 Advantages of minocycline and its lengthened therapy

Minocycline is a second-generation, semisynthetic, long-acting oral tetracycline analog that exhibits the greatest antimicrobial activity among all tetracyclines. In the past, because of its high cost and side effects, it was not used on a large scale. However, with technological progress, further improvements have been made in the price of minocycline and its pharmacokinetic properties, such as its long half-life, excellent tissue penetration, and nearly complete bioavailability, when administered orally, even in the older population. Furthermore, it is safe and well-tolerated in humans when used as a long-term treatment, even at daily doses of up to 200 mg, which is the highest dose recommended by the US FDA. The major side effects of minocycline are characteristic of the tetracycline class: gastrointestinal reaction, photosensitivity, some other central nervous system effects (eg, dizziness, nausea, vomiting, ataxia, and vertigo) and, rarely, hepatotoxicity, nephrotoxicity, and hypersensitivity.^[28]^ Overall, these adverse drug reactions mainly occur shortly after its administration and disappear rapidly after therapy discontinuation.^[29,30]^ Also, the safety of minocycline has been reviewed in detail previously.^[31]^ However, due to its teratogenic risk and inhibition effect of fetal bone growth, it is strictly prohibited to use minocycline for pregnant women. Furthermore, because it can cause permanent pediatric tooth discoloration, enamel dysplasia, and inhibit growth and development of bone, children under 8 years of age should not be treated with minocycline.^[30]^ Some research has shown that syphilis patients treated with minocycline, which was initially administered orally at 200 mg, followed by 100 mg twice daily for a limited period of 15 days, achieved satisfactory serological responses and symptom clearance for as long as 1 year; in addition, these patients tolerated the treatment well and did not experience major unpleasant side effects.^[32]^ This therapeutic strategy can even be useful for symptomatic neurosyphilis.^[33]^

Our findings generally support that the standard/lengthened minocycline and BPG therapies had similar serological cure rates in early syphilis. In addition, the serological cure rate of the lengthened minocycline therapy group was significantly higher than that of the standard therapy group under specific conditions, that is, when the patients were >40 years old, had an initial titer RPR ≥1:32, or had secondary syphilis. The long minocycline treatment course essentially extends the in vivo pharmaceutical effect and maintains an effective plasma concentration for a long time, thus eliminating the *Tp*. These findings demonstrate that minocycline is an efficacious alternative treatment for patients who are intolerant to penicillin G. Although the serological cure rate of the standard therapy group was lower than that of the lengthened therapy group; the difference was not significant and may be related to the small sample size of the study. We propose that the course of treatment for syphilis be extended, and also the duration of the follow-up period. These changes could effectively improve the cure rate of syphilis and prevent the occurrence of late syphilis.

### 4.3 Limitations

The view presented in this study had no conclusive evidence until now, as research discussing the treatment of syphilis with minocycline is rare. Moreover, we cannot ensure the compliance of all patients with oral drug regimens. In addition, because of a lack of data, our surveillance study excluded both pregnant and HIV-positive patients, which limits the generalizability of the results. Treating pregnant patients for syphilis with minocycline was forbidden because of the teratogenic risk; in addition, the treatment of syphilis patients coinfected with HIV remains unevaluated. Additional considerations include the limited research data and short follow-up periods. Therefore, multicenter experimental studies of large sample sizes over long periods are required to explore this subject further.

## 5 Conclusions

Minocycline appeared to be an effective agent for treating early syphilis because the minocycline treatments, particularly the 4-week therapy, led to high serological cure rates that were similar to that of penicillin.

## Acknowledgment

We would like to thank the Tianjin Medical University General Hospital staff for coordinating our access to the extracted data.
